# Blink-related EEG activity measures cognitive load during proactive and reactive driving

**DOI:** 10.1038/s41598-023-46738-0

**Published:** 2023-11-08

**Authors:** Emad Alyan, Stefan Arnau, Julian Elias Reiser, Stephan Getzmann, Melanie Karthaus, Edmund Wascher

**Affiliations:** Department of Ergonomics, Leibniz Research Centre for Working Environment and Human Factors, 44139 Dortmund, Germany

**Keywords:** Cognitive neuroscience, Attention, Cognitive control, Visual system

## Abstract

Assessing drivers’ cognitive load is crucial for driving safety in challenging situations. This research employed the occurrence of drivers’ natural eye blinks as cues in continuously recorded EEG data to assess the cognitive workload while reactive or proactive driving. Twenty-eight participants performed either a lane-keeping task with varying levels of crosswind (reactive) or curve road (proactive). The blink event-related potentials (bERPs) and spectral perturbations (bERSPs) were analyzed to assess cognitive load variations. The study found that task load during reactive driving did not significantly impact bERPs or bERSPs, possibly due to enduring alertness for vehicle control. The proactive driving revealed significant differences in the occipital N1 component with task load, indicating the necessity to adapt the attentional resources allocation based on road demands. Also, increased steering complexity led to decreased frontal N2, parietal P3, occipital P2 amplitudes, and alpha power, requiring more cognitive resources for processing relevant information. Interestingly, the proactive and reactive driving scenarios demonstrated a significant interaction at the parietal P2 and occipital N1 for three difficulty levels. The study reveals that EEG measures related to natural eye blink behavior provide insights into the effect of cognitive load on different driving tasks, with implications for driver safety.

## Introduction

The annual global mortality rate from automobile accidents is a staggering 1.35 million lives lost^1^. Reports indicate that the leading cause of these collisions is human error, which account for 60% of all accidents^2^. Out of these, 95% of the accidents are due to hazardous driving behaviors of the drivers themselves. Similarly, the National Highway Traffic Safety Administration has reported that 94% of crashes are caused by drivers, according to^3^. However, poor driving decisions are not the only contributing factors to accidents. Other factors like drug use or health problems can also cause accidents^4^. Therefore, continual monitoring of automobile drivers and predicting their driving performance before and during hitting the road is urgently required. This critical measure could help in saving numerous lives every year.

Neurophysiological measures can be a promising approach to gaining insights into the cognitive mechanisms underlying successful car driving and assessing the state of drivers while on the road. The electroencephalogram (EEG) allows for exploring human information processing and cognitive functioning with a high temporal resolution. It can provide information about cognitive states and task load, even in situations where no observable behavior can be recorded. Numerous investigations utilized the EEG to explore various aspects of driving performance, encompassing areas such as the incidence of mind-wandering^5^, distraction in driving^6^, and the prediction of driver drowsiness^7^ as well as other related factors^8,9^. However, these studies rely on adding event markers based on repeated stimulus presentation, which is highly artificial and not easy to realize in real driving situations, impacting the ecological validity of the driving scenario itself^10^. This also poses a challenge in naturalistic settings where visual inputs constantly change.

In recent studies, eye blinks have emerged as a promising tool for detecting cognitive load in real-life situations involving sustained and uninterrupted task performance^11–14^. Participants naturally generate eye blinks, and their detection through EEG requires no additional equipment, providing a non-intrusive and uncomplicated approach to identifying noteworthy events. Several studies have consistently shown that eye blinks serve a functional purpose in segmenting meaningful periods of visual information processing, rather than occurring randomly. This is evident in various tasks and stimuli, such as blinks commonly occurring at the end of a sentence during reading^15^, after a scene while watching movies^16^, or after a decision has been made^13^. The consistent timing of these blinks strongly suggests that they indicate the completion of processing a chunk of information. Studies on blink event-related potentials (bERPs) have revealed their responsiveness to a range of factors, including the experimental context and the specific nature of the ongoing task^14,17^. A recent study conducted in our laboratory by Wascher et al.^11^ employed blink patterns to partition continuous EEG data and deduce cognitive loads during walking tasks. The study found that blink-associated EEG signals differentiate various degrees of cognitive demand while walking. Notably, the amplitude of the parietal P3 and fronto-central N2 decreased as the demands of walking increased. Subsequently, a follow-up study also utilized eye blink-related EEG activity to examine cognitive demands during a power-plant operator simulation^18^. The findings suggest that greater task complexity is linked to increased cognitive effort and engagement, as evidenced by changes in posterior N1 and P3 amplitudes, frontal theta power, and parietal alpha power. In related experiments involving driving, a significant N1 component amplitude reduction occurred with increased task difficulty, suggesting cognitive resources were redirected to handle heightened cognitive load during challenging driving. Furthermore, Cheng et al.^19^ conducted a study using eye blink-related EEG data to evaluate cognitive load during mobile map-assisted navigation tasks. Their findings indicated that the display of five landmarks on maps enhanced spatial learning without excessively burdening cognitive resources. Furthermore, additional research has revealed that blink-evoked potentials can be affected by the cognitive demands of daily tasks, including workplace simulations^20^ and pedestrian navigation within urban environments^21^.

However, additional research is required to confirm the practicality of utilizing eye blink-related EEG activity as indicators of cognitive load and to gain deeper insights into the cognitive processes that occur after eye blinks. This will provide proof of concept for using blink-related EEG signals as a viable approach to assessing cognitive demands in various contexts. To this end, our study investigated the cognitive load of participants engaged in a simulated driving scenario while performing either a reactive or proactive driving task. The proactive and reactive driving in real-world situations require steering actions to navigate challenging scenarios, such as driving on curved roads requiring advance planning, while sudden impacts of strong crosswinds may hinder adequate preparation and compromise anticipation. The reactive driving task required reacting to external stimuli, and participants may have relied more on reactive action-control mechanisms. In contrast, the proactive driving task allowed participants to intentionally adapt to upcoming events and engage in preparatory activities^22^, resulting in a more proactive allocation of cognitive control resources. The contradictory relationship between the reactive and proactive driving tasks can be explained by the inherent differences in task demands, the controllability of the situations, and the availability of resources involved in cognitive control. These variables shaped participants’ decision-making processes and action strategies, leading to distinct outcomes in the two experiments.

Furthermore, it is assumed that the blink-related posterior P3 and anterior N2 components could be valid markers of resource allocation, task demand, and cognitive control during cognitive processing^11,18^. These components provide valuable insights into the dynamic nature of cognitive processes, with the increase in P3 amplitude in parieto-occipital regions being specifically associated with higher cognitive load^11,18^. Additionally, previous studies found that higher alpha activity could be associated with mind-wandering^23^ or an attentionally disengaged mental state. Also, boring and repetitive tasks showed this as the case^20,24,25^.

Our present investigation aimed to determine how the brain responds to blinking under different driving conditions (proactive and reactive) and difficulty levels (low, middle, and high) for each. To achieve this, we measured bERPs and spectral perturbation (bERSP) to gauge cognitive load across various driving conditions. We hypothesized that the capacity to manage the driving route proactively would result in considerable variations in posterior N1 and P3 amplitudes across different difficulty levels. This is because the management of the driving route involves the use of attentional processes, which augment the visual information presented at the sensory component. The posterior N1 component, typically corresponding to early visual perception, indicates initial encoding and processing of visual information related to driving. Proactive route management would require more detailed processing of this visual information as task difficulty increases, which could lead to modulation of N1 amplitudes. The posterior P3 component, on the other hand, is known to be involved in cognitive processes such as attentional allocation and decision making, and it could exhibit amplitude fluctuations because of dynamic allocation of cognitive resources during road navigation. Tracking changes in posterior N1 and P3 amplitudes could therefore provide a comprehensive understanding of how people modify their visual and cognitive processing strategies to cope with different levels of road complexity. Furthermore, we speculated that an upsurge in task engagement could trigger a reduction in alpha activity. Based on the assumption that the crosswinds tasks may involve distinct learning experiences that require equivalent attentional resources and visual information, regardless of the reactive driving difficulty level, we predicted that there would be no significant differences in bERPs or bERSP across the various levels of difficulty.

## Methods

### Participants

A total of 28 participants were recruited for each of the reactive and proactive driving experiments, consisting of 14 females and 14 males. These 28 participants per experiment were retained after the exclusion of 4 participants from the initial pool of 32 for each experiment, due to insufficient data quality and the challenge in accurately quantifying blinking behavior. Notably, some portions of the data from these experiments have already been published under different analyses and methodologies in^26,27^. In the present study, the reactive group had an average age of 43.3 years, with a standard deviation of 20.3, while the proactive group had an average age of 42.0 years, with a standard deviation of 20.1. All participants had been using a car at least twice a week for the past 3 years. Before being admitted to the study, potential participants were screened for eligibility based on various requirements, including the absence of neurological or psychiatric disorders, no use of substances affecting the central nervous system, and normal or corrected vision and hearing. Each participant gave their informed written consent and received a compensation of 10€ for every hour spent in the study. The study received approval from the local ethics committee of the Leibniz Research Centre for Working Environment and Human Factors and was conducted in compliance with the Declaration of Helsinki.

### Procedure and stimuli

**Figure 1:**
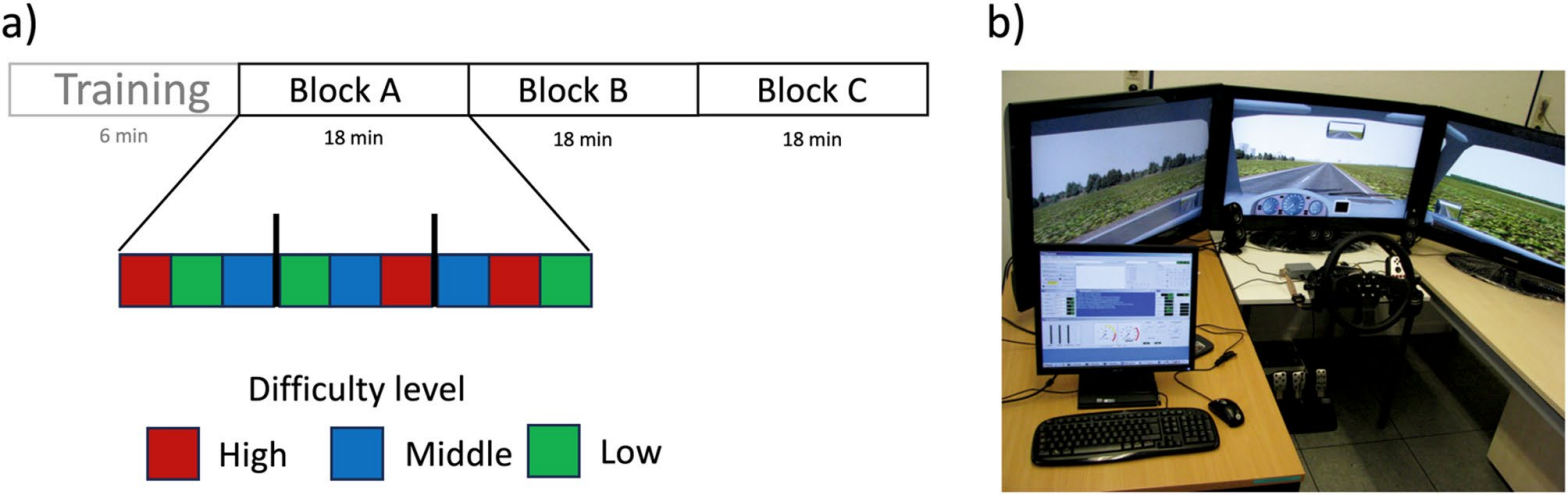
Experimental setup and task configuration. (**a**) Illustration of the task arrangement comprising an initial training block followed by three distinct experimental phases. Each experimental block comprises nine segments, each involving three difficulty levels for both reactive and proactive driving. (**b**) Depiction of the driving simulator arrangement.

The study utilized a stationary driving simulator (ST Sim; ST Software B.V. Groningen, Netherlands, Fig. 1b) to collect data, as fully detailed in^26,27^. The experiment included two driving conditions. In the first, called reactive driving, participants drove on a straight two-lane road while experiencing simulated crosswinds. A sinusoidal lateral force was generated systematically by varying the road slopes. This simulated lateral force replicated the effects of crosswinds by constantly shifting the vehicle’s position to the left and right using a complex signal composed of eight different superimposed and phase-delayed sine waves (at frequencies of 1/25.6 Hz, 1/17 Hz, 1/12.8 Hz, 1/10.2 Hz, 1/8.6 Hz, 1/7.2 Hz, 1/6.4 Hz, and 1/5.6 Hz). In the second condition, proactive driving, the participants were required to drive on a single-lane road with varying radii of curves. Both situations were set up in a plain grassy area with no additional visual distractions. The speed remained constant at 31 mph throughout. Participants were given clear instructions to keep the vehicle under precise control within the designated track. To manipulate the task difficulty, the crosswind amplitude and the curves’ radii were adjusted, with three task load levels (low, middle, and high) presented in 2-min segments combined into triplets in a randomized order (see Fig. 1a). The participants were instructed to maintain their driving accuracy (i.e., keeping the lane as precise as possible). Short transfer intervals were introduced before each task load segment to avoid abrupt shifts. The experimental design consisted of 10 sets of three task load levels. The first set served as a training session lasting 6 min, during which participants became acquainted with the experimental task. The entire experimental session lasted for 54 min without interruption.

### EEG data acquisition and processing

EEG data were recorded using a Biosemi active system, Active two, BioSemi, NL, which consisted of 64 scalp electrodes. The electrode placement was determined according to the International 10–10 system, with an additional two electrodes placed on the mastoids, located on the left and right sides. The system utilized a 2-wire active electrode approach, which adhered to the Common Mode Sensing and Driven Right Leg (CMS/DRL) principle. The data was sampled at a frequency of 2048 Hz and had a bandwidth of DC-140 Hz, while ensuring that the electrode impedance remained below 10 kΩ.

The data analyses were conducted using custom EEGLAB scripts in MATLAB^28^. The raw EEG data was subjected to finite impulse response (FIR) filtering using eegfiltnew to remove environmental and muscular artifacts. A high-pass filter with a cutoff frequency of 0.1 Hz and low-pass filter with a cutoff frequency of 40 Hz were applied. The clean_artifacts function was then applied to detect and label any bad channels with default settings (flatline = 0.5 s, burst = 5, line noise = 4, correlation = 0.8, and window = 0.25). The data was re-referenced to the common average before applying a high-pass zero-phase Hamming window FIR filter with a cutoff frequency of 1.5 Hz. The filtered data was then decomposed into statistically independent components (ICs) using AMICA^29^. Finally, the obtained ICs were copied to the average referenced data, which represents the data before high-pass filtering.

During the preprocessing stage of EEG data, blink detection is performed using the EEG BLINKER^30^. This algorithm identifies potential blinks in EEG signals that exceed 1.5 standard deviations above the mean, have a minimum duration of 50 ms, and have at least 50 ms between blinks. The algorithm computes linear fits to determine how closely the potential blink matches a typical blink and excludes blinks with blink amplitude ratios outside of 3–50. However, although the BLINKER algorithm selects the optimal blink IC by comparing the difference between the means of frontal and rear hemisphere distributions of each component, it may not always identify the most relevant eye blink IC as it may not detect blinks from saccade-related ICs. To address this issue, a modified version of the BLINKER algorithm was implemented to enhance the IC selection process. This modification involves calculating the Pearson correlation between the left and right anterior channels of eye ICs, which are classified by the IClabel algorithm^31^. The blink peaks of the continuous blink IC time series with the highest positive correlation are then selected as corresponding event markers in the EEG.event data structure to segment the data.

After decomposing the signal, the IClabel algorithm automatically classifies components and removes any ICs unrelated to the brain. This involves discarding components with classification probabilities of less than 30% for the brain and greater than 30% for the eye, muscle, heart, channel, and other classifications. The resulting data were then downsampled to 256 Hz. Epochs were extracted from − 800 ms to 1200 ms relative to the peak of the eye blink. Finally, an automatic epoch rejection function called “pop_autorej” from EEGLAB was used to discard epochs with fluctuations exceeding an absolute threshold value of 500 µV and a standard deviation threshold of 5, using an iterative approach with a maximum rejection of 10% per iteration.

### Data analysis

#### ERPs

In this study, we performed baseline correction on each subject's data using a pre-interval ranging from − 300 to − 100 ms relative to the maximum blink. We then constructed a grand average waveform by averaging the ERP waveforms across all participants, driving conditions (proactive or reactive), and difficulty levels (low, middle, and high). This approach effectively captured the common brain response to blinks while minimizing the effects of individual variability and random noise. To quantify the ERP components of interest, we measured the peak latency at the maximum for positive components or minimum for negative components within the predefined time window of each component on the grand average waveform. For example, we identified the N1 component between 70 and 150 ms and the P3 component between 250 and 400 ms. We then averaged the data specific to each subject within a time frame of ± 20 ms surrounding these peaks for each driving condition and difficulty level. To examine the impact of the conditions on the amplitude of different ERP components, we assessed various sites, including frontal (AF3, Fz, and AF4), parietal (Pz), and occipital (PO3, Oz, and PO4). This analysis allowed us to investigate the effects of different driving conditions and difficulty levels on the brain's response to blinks.

#### Time–frequency power

The present study utilized the Morlet wavelet transformation method to analyze blink-related spectral perturbations in EEG power over time. This method involves the computation of the similarity between the input signal and Gaussian-windowed complex sinusoidal functions, and is a widely accepted and reliable approach for investigating neural processes underlying EEG data^32,33^. The frequency range selected for this analysis ranged from 2 to 35 Hz, encompassing 70 frequencies. Wavelet cycles between 3 and 10 were employed to ensure both temporal and frequency accuracy. To quantify the changes in the temporal and spectral aspects during the reactive and proactive difficulty levels, the data at each time point were normalized by dividing it with the mean power spectra in the pre-blink period (− 400 to − 200 ms) at each frequency for each participant across all trials. The resulting values are then transformed into decibels (dB) by calculating their base-10 logarithm and multiplying the result by 10. The analysis focused on determining the difference in the average power across all electrodes, and the power series for three frequency bands, namely theta (4 − 7 Hz), alpha (8 − 13 Hz), and beta (14 − 28 Hz), were then analyzed.

### Statistical analysis

To assess the impact of reactive and proactive driving conditions at different task levels, as well as their interaction on cognitive load, we performed a linear mixed-effects (LME) model to examine the effect detected by the ERP amplitudes. This model was constructed using the MATLAB fitlme function, which fitted the data using the restricted maximum likelihood (REML). The subject served as the random effect factor, signifying that the effect could vary between subjects. The conditions of reactive or proactive driving and levels of task difficulty, as well as their interaction (Condition * Level), served as fixed effect factors. Two models were developed; one includes either proactive or reactive driving conditions at various task levels is given by Eq. 1, while the second model represents the interaction between these conditions and levels, and is described by Eq. 2:

1 $${\text{Amplitude }}\sim { 1 } + {\text{ Level }} + \, \left( {{1 }|{\text{ Subject}}} \right)$$

2 $${\text{Amplitude }}\sim { 1 } + {\text{ Condition}}*{\text{Level }} + \, \left( {{1}|{\text{Subject}}} \right)$$

where amplitudes are the ERP amplitudes. The term “Level” stands for low, middle, and high task difficulty levels. Condition reflects proactive and reactive driving conditions. To calculate Cohen’s d^34,35^, which represents standardized effect sizes for each fixed-effect coefficient, we utilized the estimate divided by its standard deviation (SD). The SD was estimated using the standard error (SE) obtained from the *fitlme* function in MATLAB. This involved multiplying the SE by the square root of the degrees of freedom (DF), which, in this instance, was 27 (DF = 28 − 1). In addition, analyses of variance (ANOVAs) were performed on the time–frequency power to investigate changes in neural activity across levels of proactive or reactive driving condition. We estimated the F-value of ANOVA for both driving conditions (proactive and reactive) using variation in frequency over time^36^. This was accomplished by dividing the variance between groups (i.e., among task levels) by the variance within the group (i.e., within the same task level). A high F-value demonstrates a small change within the same condition and a large difference between conditions.

## Results

### Blink-related ERPs

**Figure 2:**
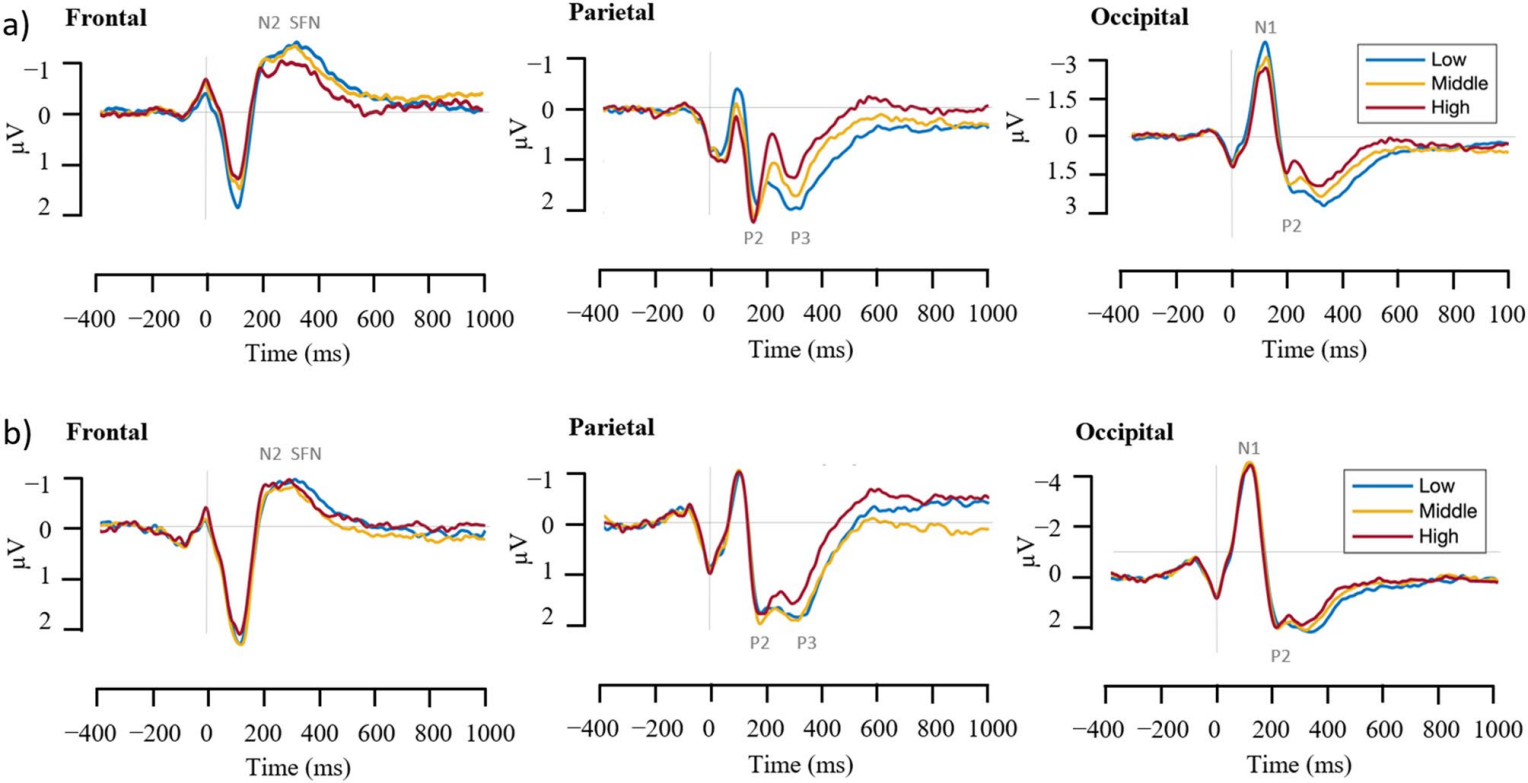
Grand-average ERP curves for the frontal (AF3, Fz, AF4), parietal (Pz), and occipital (PO3, Oz, PO4) brain areas for (**a**) proactive and (**b**) reactive driving with three task difficulty conditions (low, middle and high). The ERPs are color-coded according to task difficulty conditions, with blue for low, orange for middle, and red for high.

**Figure 3:**
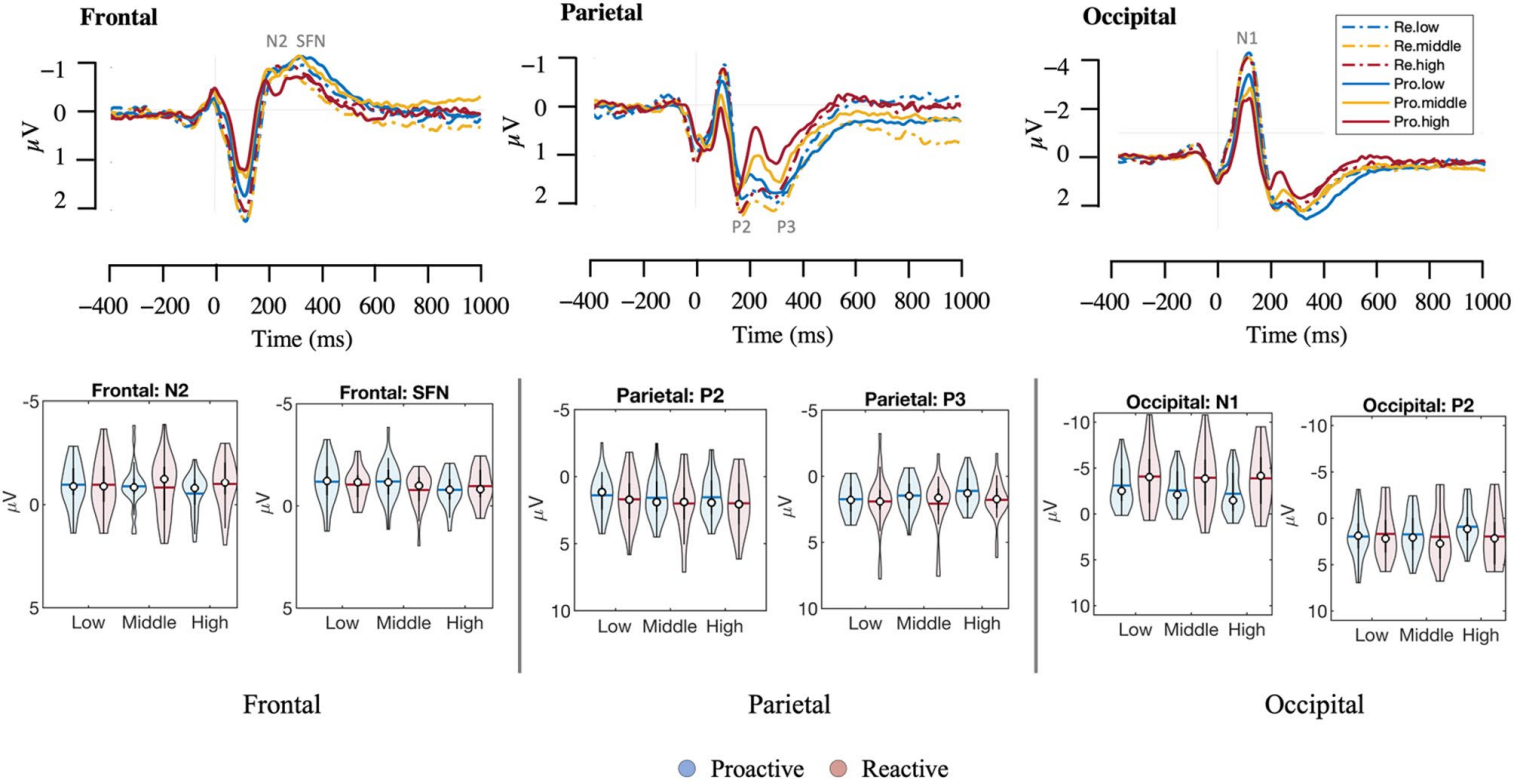
Top panel: Blink-locked ERP changes in three task difficulty conditions during reactive (dashed line) and proactive driving (solid line) at the frontal (AF3, Fz, AF4), parietal (Pz), and occipital (PO3, Oz, PO4) brain areas. The task difficulty conditions are color-coded in blue for low, orange for middle, and red for high. Bottom panel: violin plots of ERP peaks across three different brain regions: frontal (N2 and SFN), parietal (P2 and P3), and occipital (N1 and P2). The blue and red lines denote the mean values, while the white cycles represent the median values.

An LME analysis was performed to determine how ERP responses differ across different levels of driving difficulty. This comprised both proactive and reactive driving, as well as their interactions across the frontal, parietal, and occipital areas (see Figs. 2 and 3). For proactive driving, the results revealed that N1 amplitude in the occipital area decreased significantly with increasing levels of driving task [t (2, 82) = 5.092, β ± SE: 0.46 ± 0.09, CIs (0.28, 0.64), *p* < 0.0001, d = 0.98], with amplitudes being larger in the low proactive driving condition compared to the middle and high driving conditions. Furthermore, increasing proactive driving complexity appeared to be associated with decreasing amplitudes of parietal P3 and occipital P2, but increasing amplitudes of parietal P2. All three components' amplitudes [parietal P3: t (2, 82) = − 3.16, β ± SE: − 0.318 ± 0.101, CIs (− 0.518, − 0.118), *p* = 0.002, d = 0.61; occipital P2: t (2, 82) = − 2.549, β ± SE: − 0.322 ± 0.126, CIs (− 0.574, − 0.071), *p* = 0.013, d = 0.49; parietal P2: t (2, 82) = 2.193, β ± SE: 0.168 ± 0.076, CIs (0.016, 0.32), *p* = 0.031, d = 0.43] varied significantly across the three levels of proactive driving conditions. The LME analysis also showed a significant reduction in frontal ERPs at N2 [t (2, 82) = 2.184, β ± SE: 0.152 ± 0.07, CIs (0.014, 0.29), *p* = 0.032, d = 0.42] and SFN [t (2, 82) = 2.422, β ± SE: 0.19 ± 0.078, CIs (0.034, 0.346), *p* = 0.018, d = 0.47] with increasing task difficulty.

On the other hand, the study found no significant differences in ERPs across all brain regions between the three levels of reactive driving conditions. Interestingly, the consistent patterns of ERP waves across different difficulty levels with proactive and reactive driving support the high reliability of these indicators when evaluating cognitive load while driving. The statistical analysis outcomes for proactive and reactive driving are fully summarized in Tables 1 and 2, respectively.

**Table 1:** Summary of statistical analysis results for ERPs in the proactive driving task.

Brain region	ERP	Latency (ms)	Predictor	β	SE	t-value	*p-*value	Cohen’s d	95% CIs	
									Lower	Upper
Frontal	N2	250	Intercept	− 1.31	0.226	− 5.783	< 0.0001	1.12	− 1.76	− 0.859
			Condition	0.152	0.07	2.184	0.032*	0.42	0.014	0.29
	SFN	324.2	Intercept	− 1.57	0.208	− 7.564	< 0.0001	1.45	− 1.983	− 1.157
			Condition	0.19	0.078	2.422	0.018*	0.47	0.034	0.346
Parietal	P2	160.2	Intercept	1.501	0.382	3.927	< 0.0001	0.76	0.74	2.261
			Condition	0.168	0.076	2.193	0.031*	0.43	0.016	0.32
	P3	300.8	Intercept	2.245	0.289	7.762	< 0.0001	1.49	1.67	2.821
			Condition	− 0.318	0.101	− 3.16	0.002**	0.61	− 0.518	− 0.118
Occipital	N1	117.2	Intercept	− 3.783	0.469	− 8.062	< 0.0001	1.55	− 4.717	− 2.85
			Condition	0.46	0.09	5.092	< 0.0001***	0.98	0.28	0.64
	P2	203.1	Intercept	2.267	0.478	4.741	< 0.0001	0.91	1.316	3.218
			Condition	− 0.322	0.126	− 2.549	0.013*	0.49	− 0.574	− 0.071

β denotes the estimates, SE is the standard error, and Cohen’s d represents the standardized effect size. The LME model analyzed data from 84 observations, which were collected from 28 participants.

*p < 0.05.

**p < 0.01.

***p < 0.001.

**Table 2:** Summary of statistical analysis results for ERPs in the reactive driving task.

Brain region	ERP	Latency (ms)	Predictor	β	SE	t-value	*p* value	Cohen’s d	95% CIs	
									Lower	Upper
Frontal	N2	246	Intercept	− 0.868	0.296	− 2.936	0.004	0.56	− 1.457	− 0.28
			Condition	− 0.001	0.065	− 0.009	0.993	0	− 0.13	0.129
	SFN	301	Intercept	− 0.938	0.197	− 4.755	0	0.92	− 1.33	− 0.546
			Condition	0.003	0.057	0.059	0.953	0.01	− 0.11	0.116
Parietal	P2	180	Intercept	1.72	0.413	4.163	0	0.8	0.898	2.542
			Condition	0.038	0.077	0.494	0.623	0.09	− 0.115	0.19
	P3	297	Intercept	2.062	0.393	5.25	0	1.01	1.281	2.843
			Condition	− 0.137	0.087	− 1.563	0.122	0.3	− 0.311	0.037
Occipital	N1	117	Intercept	− 4.246	0.607	− 6.998	0	1.35	− 5.453	− 3.039
			Condition	0.066	0.056	1.191	0.237	0.23	− 0.045	0.177
	P2	215	Intercept	1.775	0.587	3.026	0.003	0.58	0.608	2.942
			Condition	0.072	0.083	0.875	0.384	0.17	− 0.092	0.237

β denotes the estimates, SE is the standard error, and Cohen’s d represents the standardized effect size. The LME model analyzed data from 84 observations, which were collected from 28 participants.

The analysis has been expanded to investigate the interaction between the conditions and the task levels for both proactive and reactive driving to gain a more comprehensive understanding of the driving conditions. This expanded analysis aimed to provide deeper insights into how different driving conditions and difficulty levels might affect overall driving behavior and performance. According to Fig. 3 and Table 3, the results revealed a significant interaction between task difficulty level and condition that had a noticeable effect on the parietal P2 [t (4, 164) = 2.002, β ± SE: 0.655 ± 0.327, CIs (0.009, 1.302), p = 0.047, d = 0.39] and occipital N1 [t (4, 164) = 3.226, β ± SE: 1.55 ± 0.481, CIs (0.602, 2.499), *p* = 0.002, d = 0.62] regions. This finding indicates that this interaction could discernibly impact the electrical activity in these brain areas, with the occipital N1 decreasing and the parietal P2 increasing with increasing difficulty of the proactive, but not reactive, driving task.

**Table 3:** Statistical analysis summary of ERPs for the interaction between proactive and reactive driving tasks.

Brain region	ERP	Latency (ms)	Predictor	β	SE	t-value	*p* value	Cohen’s d	95% CIs	
									Lower	Upper
Frontal	N2	250	Intercept	− 2.684	0.735	− 3.651	0	0.7	− 4.135	− 1.233
			Level	0.809	0.34	2.379	0.019*	0.46	0.138	1.481
			Condition	0.756	0.465	1.627	0.106	0.31	− 0.162	1.674
			Level * Condition	− 0.31	0.215	− 1.44	0.152	0.28	− 0.735	0.115
	SFN	313	Intercept	− 0.974	0.579	− 1.68	0.095	0.32	− 2.118	0.171
			Level	0.085	0.268	0.318	0.751	0.06	− 0.444	0.615
			Condition	0.005	0.366	0.013	0.989	0	− 0.719	0.729
			Level * Condition	− 0.065	0.17	− 0.385	0.701	0.07	− 0.4	0.27
Parietal	P2	168	Intercept	4.178	1.118	3.736	0	0.72	1.969	6.386
			Level	− 0.945	0.518	− 1.825	0.07	0.35	− 1.967	0.077
			Condition	− 1.702	0.707	− 2.407	0.017*	0.46	− 3.099	− 0.306
			Level * Condition	0.655	0.327	2.002	0.047*	0.39	0.009	1.302
	P3	301	Intercept	4.004	1.012	3.958	0	0.76	2.006	6.001
			Level	− 0.801	0.468	− 1.71	0.089	0.33	− 1.725	0.124
			Condition	− 1.324	0.64	− 2.069	0.04*	0.4	− 2.587	− 0.06
			Level * Condition	0.42	0.296	1.418	0.158	0.27	− 0.165	1.005
Occipital	N1	117	Intercept	− 0.109	1.641	− 0.066	0.947	0.01	− 3.35	3.133
			Level	− 2.602	0.76	− 3.424	0.001**	0.66	− 4.102	− 1.102
			Condition	− 1.752	1.038	− 1.688	0.093	0.32	− 3.802	0.297
			Level * Condition	1.55	0.481	3.226	0.002**	0.62	0.602	2.499
	P2	211	Intercept	3.932	1.635	2.404	0.017	0.46	0.703	7.161
			Level	− 0.85	0.757	− 1.123	0.263	0.22	− 2.345	0.645
			Condition	− 1.186	1.034	− 1.147	0.253	0.22	− 3.229	0.856
			Level * Condition	0.421	0.479	0.879	0.38	0.17	− 0.524	1.367

β denotes the estimates, SE is the standard error, and Cohen’s d represents the standardized effect size. The LME model analyzed data from 168 observations, which were collected from 28 participants.

*p < 0.05.

**p < 0.01.

***p < 0.001.

Additionally, the overall condition main effect demonstrated a statistically significant difference in the parietal ERP components between the two conditions. Particularly, P2 and P3 amplitudes were significantly higher [P2: t (4, 164) = − 2.407, β ± SE: − 1.702 ± 0.707, CIs (− 3.099, − 0.306), *p* = 0.017, d = 0.46; P3: t (4, 164) =  − 2.069, β ± SE: − 1.324 ± 0.64, CIs (− 2.587, − 0.06), *p* = 0.04, d = 0.4] in reactive driving than in proactive driving. It is important to note that the main effect of the overall condition did not reveal any discernible differences in the ERP components in the frontal or occipital regions, suggesting that the effects are restricted to the parietal region.

The main effect of task level on brain electrical activity has been noticed through significant changes in negative ERP components at the frontal N2 [t (4, 164) = 2.379, β ± SE: 0.809 ± 0.34, CIs (0.138, 1.481), *p* = 0.019, d = 0.46] and occipital N1 [t (4, 164) =  − 3.424, β ± SE: − 2.602 ± 0.76, CIs (− 4.102, − 1.102), *p* = 0.001, d = 0.66] regions. Specifically, as the level of task difficulty or complexity increases, the amplitude of negative ERP components in these areas decreases, indicating that task difficulty level significantly impacts the electrical activity of these brain areas.

### Blink related ERSPs

**Figure 4:**
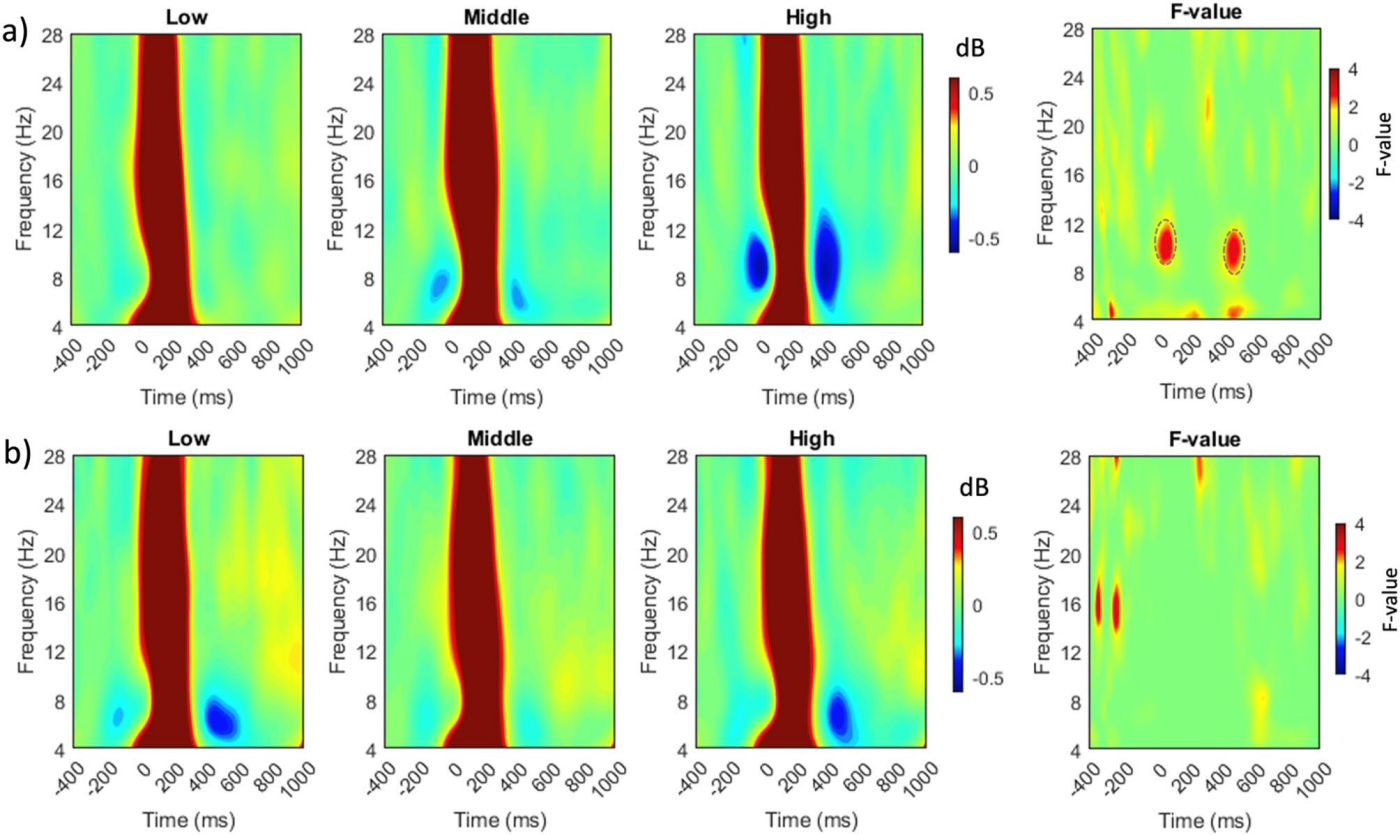
Averaged time–frequency power spectra in response to blink-related activity during (**a**) proactive and (**b**) reactive driving at three different levels of difficulty (low, middle, and high) with a frequency range of 4–28 Hz. The plots depict the average data obtained from all electrodes. The right-most panels in (**a**) and (**b**) exhibit the F-value of ANOVA in time–frequency analysis, with dashed ovals indicating significant time–frequency windows.

The average time–frequency power of low, middle, and high levels of proactive and reactive driving is shown in Fig. 4a,b across all electrodes and subjects. The reference point, time zero, reflects the peak of the blink. Blue indicates power reduction, whereas red indicates power increase relative to the baseline. To detect neural characteristics that reflect conditional variations, the F-value ANOVA of ERSPs data was calculated, as displayed in the panels on the most-right of Fig. 4a,b for proactive and reactive driving, respectively. These findings revealed significant differences between proactive and reactive driving scenarios. Significant differences were observed in two clusters within the alpha band (8–13 Hz) in the proactive driving condition. These clusters occurred approximately between − 40 and 120 ms and between 400 and 500 ms relative to the blink peak. On the other hand, in the reactive driving condition, no significant variations were found following a blink. However, two significant tiny clusters were detected in the lower beta band prior to the onset of the blink, possibly implying motor processing during reactive driving.

**Figure 5:**
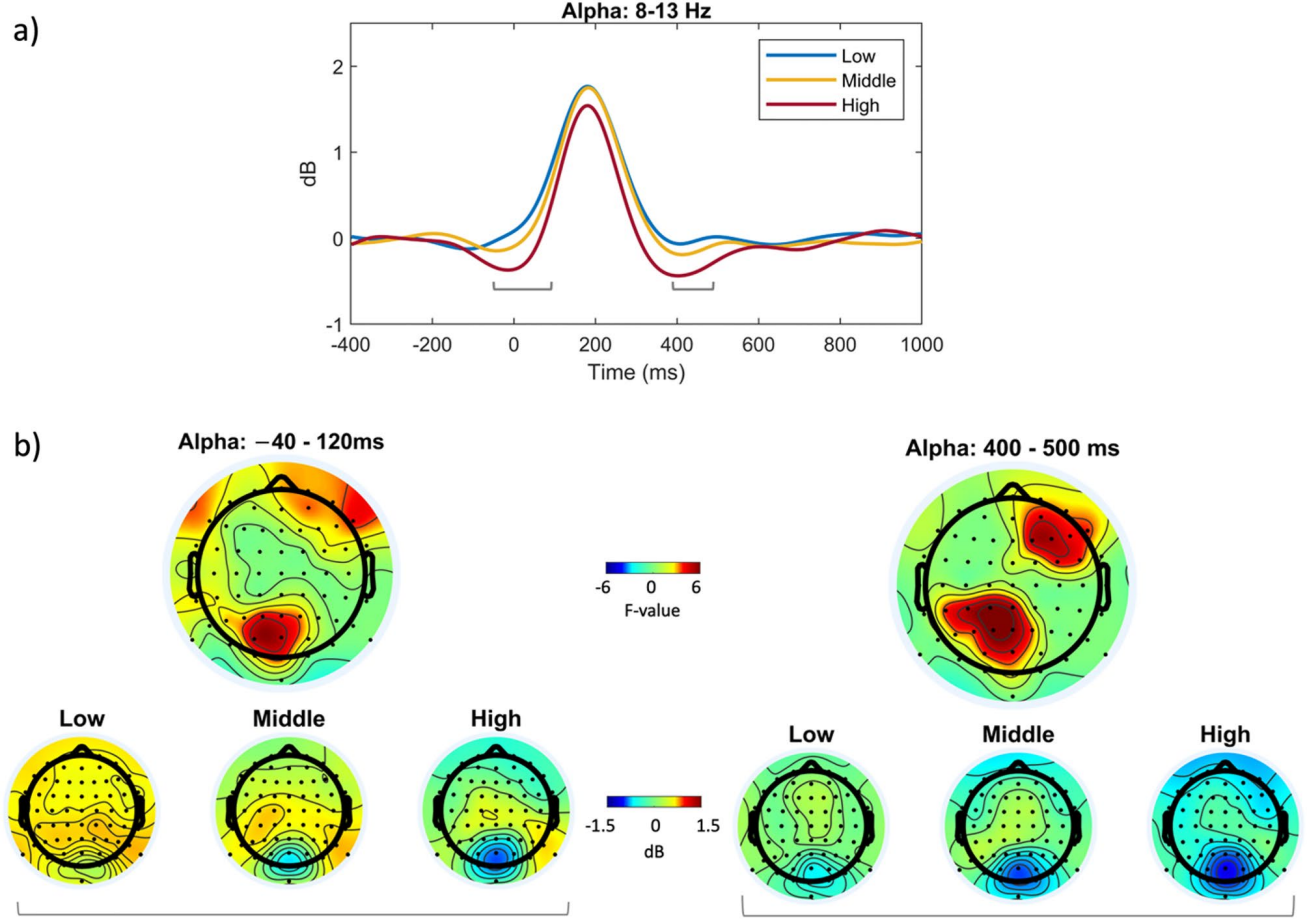
Grand average results for the time frequency analysis at the alpha band (8–13 Hz). Panel (**a**) displays a two-dimensional plot of the alpha frequency range for all three conditions. The amplitudes of the two highlighted clusters in Fig. 4a are pointed with brackets. The top row in panel (**b**) depicts F-value topographies of the early (left) and late (right) cluster, while the bottom row shows alpha power topographies for the early-to-blink cluster (averaged within the − 40 to 120 ms) and late-from-blink cluster (over 400–500 ms), respectively.

To explore the effects of the significant clusters highlighted in Fig. 4 on brain regions, these clusters were further analyzed in detail. The mean power of alpha (8–13 Hz) frequencies was determined to closely track the impact of the two clusters across the three-level conditions, as displayed in Fig. 5. The signal power of the alpha rhythm, underlined by brackets indicating two major cluster sites, as shown in Fig. 5a, exhibits a decrease in alpha power as the complexity level of proactive driving increases. This described effect is clearly localized in the parietal-occipital and right frontal regions, as shown in Fig. 5b.

## Discussion

The current study investigated cognitive load, as determined by EEG, during three levels (low, middle, and high) of proactive and reactive driving in simulated driving settings. Changes in cognitive load during driving were assessed using blink-related ERPs and ERSPs. During proactive driving, the bERPs exhibited notable amplitude variations across different task difficulty levels. The variations were particularly evident in the N2 and SFN at the frontal leads, the P2 and P3 at the parietal leads, and the N1 and P2 at the occipital leads. These ERP components exhibit traits consistent with previous ERP studies^11,18^. We hypothesized that tasks involving crosswinds, irrespective of their varying levels of reactive driving difficulty, would demand similar attentional resources and visual information. In line with these expectations, our findings indeed demonstrated that none of the examined ERP components exhibited a significant impact during reactive driving conditions. This outcome can be attributed to the automatic nature of the responses needed to preserve vehicle control. The occurrence of crosswind and its strength were not predictable and the (steering) effort to compensate for it was therefore not foreseeable. Thus, the varying degrees of unexpected crosswinds caused by different reactive driving levels may not necessitate a significant difference in attention or cognitive processing. On the other hand, proactive driving demands more cognitive effort and attention to predict possible risks, resulting in a significant effect on blink-related ERPs. This suggests that drivers were actively processing information about steering complexity, expecting potential hazards, and adjusting their driving behavior accordingly.

The most notable difference among proactive driving level conditions, with a substantial effect size, was found in the occipital N1, indicating that varying levels of steering complexity may affect drivers' sensory visual perception. This effect is closely tied to the attentional requirements of the specific driving situation, aligning with previous research that studied blink-related ERPs^11,14,18^. For instance, Alyan et al.^18^ revealed that posterior N1 components are sensitive to task requirements, indicating that the brain adapts to selective adjustments of visual demands. This is demonstrated by a higher N1 amplitude during passive steam engine operation compared to active steam engine operation. Moreover, as the steering complexity increased, a significant decrease in the amplitudes of higher-level components, such as the frontal N2, parietal P3 and occipital P2, was seen. The amplitude dropped, indicating that as the brain processed more complex input, attention focused more on relevant information^11,37^. It could also be interpreted as reducing mental resource availability as task complexity increased^38^. Similarly, this phenomenon has been noticed in the SFN (N300) amplitude, demonstrating the perceptual characteristics of visual input^39,40^. Additionally, there was a noticeable increase in the parietal P2 amplitude, consistent with an increased attentional focus^41,42^, indicating that more cognitive resources were needed to distinguish between pertinent information during highly proactive driving.

We also found a significant interaction effect between the proactive and reactive driving conditions with the three difficulty levels at the occipital N1 and parietal P2 components. The significant interaction effect implies that the processing of visual information and attention allocation in relation to the various driving conditions varied depending on the difficulty level. This suggests that the cognitive and perceptual demands of the two driving conditions differed, leading to distinct impacts on the participants' neural processing. Although the proactive condition had a strategic attention orientation, a stronger N1 was seen in the reactive condition. This finding aligns with the previous research conducted by Wascher et al.^11^ which reported a decreased occipital N1 response during traversing an obstacle course with high attentional demands, compared to walking. Using a selective stop signal task, Raud et al.^43^ found that reactive control had a larger N1 than proactive control, suggesting greater attentional processing of the stop signal in the reactive control, while a more sustained and prepared attentional state in the proactive control. Therefore, the high task demands required during reactive driving may explain the higher N1 reported in the reactive condition as participants had to react to sudden crosswinds, which called for quick shifts in attention and more visual processing resources to determine the direction and force of the wind. As the brain must devote more resources to process visual information effectively, this increased demand for attentional resources may result in a stronger N1 component. On the other hand, participants in the proactive condition may have had to engage more planned efforts to prepare for and react to the steering difficulty levels. This might have resulted in a diminished N1 since the brain would need to allocate resources to strategic processing over early visual processing. The significant main effects of the condition on the parietal P3 amplitude support this conclusion. The parietal P3 indicates attentional resource allocation^11^, with a smaller P3 amplitude implying that less attentional resources are being directed to the activity. As a result, the reported decrease in P3 amplitude during proactive versus reactive driving lends credence to the notion that proactive driving uses less (and more adaptively used) attentional resources than reactive driving.

The study found high similarity between the bERPs and bERSP results. These revealed a significant difference between task levels of proactive driving, but not between task levels of reactive driving. The alpha power over brain regions decreases as the task load of proactive driving increases, specifically in two clusters between  − 40 and 120 ms and 400 and 500 ms relative to blink peak. These clusters could be related to the onset and offset effects of the visual scene. The presence of the alpha frequency has been linked to a state of “wakefulness” in the brain, as it becomes less synchronized during cognitive activities^44,45^. The decrease in alpha power reveals that the driver is shifting their attention towards the visual scene and preparing to react to potential hazards. This implies that the brain devotes more resources to the task and less to background activity. Furthermore, higher alpha activity has been linked to a state of attentional withdrawal^5^, so a decrease in alpha power might signify that more cognitive resources are being allocated to the task, resulting in greater neural activity and higher alpha suppression, particularly observed in the parieto-occipital regions. These brain regions are more engaged during demanding tasks requiring increased attention and cognitive resources^46,47^. This concept aligns with the "inhibition-timing hypothesis" proposed by Klimesch et al.^48^, which states that alpha synchronization could be linked to an active mechanism for suppressing irrelevant information during a task. According to this hypothesis, when individuals encounter a cognitive challenge, cortical areas involved in task processing experience a reduction in alpha power, while areas not essential to the task or potentially interfering with it are inhibited through alpha synchronization, particularly in the parieto-occipital regions^48,49^.

The findings are consistent with our hypotheses, furnishing compelling evidence that the ability to anticipate a driving route does give rise to perceivable variations in neural responses during proactive driving, particularly in posterior N1 and P3 amplitudes. They also support the notion that reductions in alpha activity correspond to heightened levels of task engagement, which remains relatively stable across varying difficulty levels. These outcomes reinforce the validity of our research framework and contribute to a comprehensive understanding of how individuals adapt their visual and cognitive processing strategies in response to varying road complexities.

Furthermore, the findings have significant implications for driver safety. Our results emphasize the importance of utilizing EEG for investigating the complexities of driver’s attentional allocation and advancing the field of driving safety research. By using blink-related potentials, we could open promising avenues for assessing cognitive load and examining safety–critical events in drivers across diverse cognitive states. This breakthrough offers the potential to revolutionize the assessment of how drivers allocate their visual attention, thereby enhancing our ability to predict and mitigate potential driving hazards. While our investigation found substantial differences in cognitive states using blink-ERPs and ERSPs, further limitations and directions should be acknowledged. One limitation of our study was the inherent inability to establish a conclusive and definitive relationship between eye-blinking and crosswind events. This limitation primarily arose from the scarcity of detailed crosswind trigger information, which hindered our ability to account for potential confounding factors arising from variations in task complexity and attentional demands. Consequently, we suggest the acquisition of detailed crosswind trigger data, encompassing precise wind conditions and pertinent parameters, to enable the development of more sophisticated experimental methodologies in the future. Additionally, the current study only looked at changes in cognitive load while driving and may not apply to other real-world settings. Although the study's findings revealed that the blink-related EEG analysis could be used as a marker of cognitive load, more research is required using real-world driving scenarios or other real-world tasks to prove their validity and reliability. In the future, integrating eye-tracking and EEG measurements may assist in producing more precise and in-depth information on the evaluation of cognitive load. Thus, although there is still a long way to go, our study provides a potential grounding for more research.

## Conclusion

The study employed EEG-measured bERPs and ERSPs to investigate the cognitive load of drivers in simulated settings at different difficulty levels (low, middle, and high) of reactive and proactive driving difficulty. The findings revealed that proactive driving required a more strategic and flexible attentional orientation, whereas reactive driving demanded a more spontaneous and unplannable attentional orientation. As a result, significant effects of steering complexity on bERPs and ERSPs were observed during proactive driving, which influenced drivers' visual perception. However, no significant differences between reactive driving levels were observed, possibly because the steering behavior needed to maintain vehicle control in response to crosswinds was more reflexive and automatic and therefore demanded constant allocation of attentional resources across all levels. Furthermore, there was a significant interaction effect with the three difficulty levels between proactive and reactive driving conditions, implying that the two driving scenarios required different levels of cognition and perception. The results of the bERP and bERSP analyses were highly comparable, indicating a decrease in alpha power as the task load of proactive driving increased, indicating more cognitive resources dedicated to the task. Overall, the study demonstrates that blink-related EEG measures offer insights into the effect of cognitive load of different driving tasks on the driver’s brain processing.

## Data Availability

The datasets generated during the current study are available from the corresponding author on reasonable request.
